# Effect of used Mango and Karanj chewing stick (Datun) on *Streptococcus mutans*: An *in vitro* study

**DOI:** 10.6026/973206300223245

**Published:** 2026-06-30

**Authors:** Soni Soni, Dhananjay Kumar, Garima Mangal, Nirmala Kumari

**Affiliations:** 1 Department of Public Health Dentistry, Buddha Institute of Dental Sciences &Hospital, Kankarbagh, Patna, Bihar, India; 2 Department of Dentistry, Darbhanga Medical College &Hospital, Darbhanga, Bihar, India

**Keywords:** Antimicrobial activity, dental caries, *Mangifera indica*, *pongamia pinnata*, *Streptococcus mutans*

## Abstract

Dental caries remains a major oral health problem, necessitating effective natural antimicrobial alternatives targeting *Streptococcus Mutans*. Chewing sticks from *Mangifera indica* and *pongamia pinnata* contain bioactive compounds with established antiseptic and bactericidal properties. Aqueous extracts (5%, 10% and 50% w/v) were prepared and tested against *Streptococcus mutans* using agar diffusion to assess zones of inhibition. *Mangifera indica* showed maximum antimicrobial efficacy at 50% concentration, followed by lower but notable effects at 10% and 5%. Thus, we show *Mangifera indica* as a potent natural anti-cariogenic agent with potential integration into preventive oral healthcare strategies.

## Background:

Oral hygiene practices have been followed since antiquity, with chewing sticks being among the earliest methods of mechanical plaque control [1]. Historical records indicate their use by Babylonians as early as 3500 BC and they remain widely used in rural parts of India as Datun. Chewing sticks such as neem and babool have been extensively studied for their antimicrobial properties against cariogenic microorganisms [2]. Dental caries is one of the most prevalent chronic diseases worldwide and *Streptococcus mutans* plays a primary etiological role in its initiation and progression [3]. Inhibition of *S. mutans* growth is considered essential in preventing dental caries. Various *in vitro* studies have demonstrated the antimicrobial effects of plant extracts, including neem and mango, against cariogenic organisms [4]. Chewing sticks contain bioactive constituents such as tannins, resins, volatile oils, sulphur compounds, sterols, fluoride and silica, which exhibit antiseptic, astringent, anti-inflammatory and bactericidal properties. These compounds contribute to plaque reduction, prevention of caries, elimination of halitosis and stimulation of gingival tissues [5]. The natural toothbrush *(Salvadora persica)* has also shown effectiveness in reducing plaque and caries incidence. Previous studies have evaluated the antimicrobial activity of neem and mango extracts against cariogenic. However, limited data are available comparing mango and karanj chewing sticks [6]. Therefore, it is of interest to assess the antimicrobial efficacy of *Mangifera indica* and *pongamia pinnata* chewing stick extracts against *Streptococcus mutans* and to compare their activity at different concentrations.

## Methodology:

An *in vitro* experimental study was conducted in a laboratory setting after obtaining ethical approval from the Institutional Ethics Committee. Small branches of *Mangifera indica* (mango) and *pongamia pinnata* (karanj) were collected locally, cut into approximately 15 cm pieces, sun-dried for seven days and ground into coarse powder. The powdered material was weighed into 5 g, 10 g and 50 g quantities and transferred into separate labeled containers containing 100 ml of sterile deionized distilled water. These mixtures were shaken and allowed to macerate for 48 hours at 4°C, followed by filtration to obtain aqueous extracts of 5%, 10% and 50% concentrations. *Streptococcus mutans* samples were procured from the Department of Microbiology, cultured on nutrient agar and incubated aerobically at 37°C for 48 hours, with colonies confirmed microscopically after staining. Antimicrobial activity was assessed using the disc diffusion method, wherein sterile Whatman No.1 filter paper discs (6 mm diameter) were autoclaved, dried and placed on agar plates inoculated with S. mutans. Extracts were applied using a micropipette, with sterile distilled water serving as the control. Plates were incubated at 37°C for 48 hours and zones of inhibition were measured in millimeters using a Vernier caliper at four perpendicular points, with mean values recorded at 24 and 48 hours. The data obtained were analyzed observationally without the application of statistical tests.

## Results:

The antimicrobial activity of both *Mangifera indica* and *pongamia pinnata* extracts demonstrated a clear concentration-dependent pattern, with higher concentrations exhibiting greater inhibitory effects against *Streptococcus mutans* (Table 1, Table 2). As shown in Table 1, *Mangifera indica* extract exhibited maximum antimicrobial activity at 50% concentration, with a zone of inhibition measuring 2.7 mm at 48 hours, while lower concentrations showed comparatively reduced effects. As illustrated in Table 2, *pongamia pinnata* extract also demonstrated antimicrobial activity in a concentration-dependent manner, with the highest inhibition observed at 50% concentration (1.8 mm at 48 hours). As summarized in Table 3 highlights that the 50% *Mangifera indica* extract exhibited superior antimicrobial efficacy compared to *pongamia pinnata*. Table 4, no inhibitory effect was observed at 5% concentration for either extract, while 10% and 50% concentrations showed progressively increasing antimicrobial activity. Overall, *Mangifera indica* demonstrated greater efficacy than *pongamia pinnata*, particularly at higher concentrations.

## Discussion:

The present *in vitro* study demonstrated that both *Mangifera indica* and *pongamia pinnata* extracts exhibit concentration-dependent antimicrobial activity against *Streptococcus mutans*, with *Mangifera indica* showing superior efficacy. These findings are consistent with recent evidence highlighting the growing role of plant-based agents in caries prevention. A recent *in vitro* study by Lilly *et al.* (2024) [7] reported that hydroalcoholic extracts of *Mangifera indica* showed significantly higher antimicrobial activity against *S. mutans*, with large zones of inhibition comparable to chlorhexidine. This strongly supports the present findings, where higher concentrations of mango extract demonstrated enhanced antibacterial efficacy, indicating a dose-dependent mechanism. Similarly, Kajjari *et al.* (2024) [8], in an *in vivo* study, found that *Mangifera indica*-based mouthwash significantly reduced *S. mutans* counts in children, confirming its clinical applicability as a preventive oral health agent. This aligns with the current study, reinforcing the translational potential of mango-derived formulations from laboratory to clinical settings. A systematic review by Salimi *et al.* (2023) [9] further emphasized that various parts of *Mangifera indica* possess potent anti-cariogenic and antimicrobial properties, making it an economical and effective alternative for caries prevention. The present results corroborate these conclusions, particularly highlighting the strong antibacterial action at higher concentrations. In contrast, limited recent studies are available specifically on *pongamia pinnata* in oral microbiology; however, earlier mechanistic evidence by Bodiba *et al.* (2018) [10] demonstrated that *pongamia pinnata* exhibits comparatively lower antibacterial activity against *S. mutans*, especially when compared to *Mangifera indica*. This observation is consistent with the present study, where karanj extract showed moderate inhibition only at higher concentrations. Additionally, recent research on herbal antimicrobials, such as Shivakumar *et al.* (2022) [11], has shown that plant-derived extracts exhibit significant inhibitory effects on *S. mutans*, primarily due to bioactive phytochemicals like flavonoids and phenolic. This supports the mechanism proposed in the current study, where similar phytoconstituents may contribute to antibacterial action.

## Conclusion:

*Mangifera indica* extract showed superior antimicrobial activity against *Streptococcus mutans* compared to *pongamia pinnata*, particularly at higher concentrations. Both extracts exhibited a concentration-dependent inhibitory effect, with no activity observed at lower concentrations. Thus, date shows the potential use of *Mangifera indica* as an effective natural anti-cariogenic agent in preventive oral healthcare.

## Figures and Tables

**Table 1:** Effect of various concentrations of *Mangifera indica* extract on *Streptococcus mutans*

**Concentration (%)**	**24 Hours (mm)**	**48 Hours (mm)**
5%	No inhibition	No inhibition
10%	0.7	1.8
50%	1.3	2.7

**Table 2:** Effect of various concentrations of *Pongamia pinnata* extract on *Streptococcus mutans*

**Concentration (%)**	**24 Hours (mm)**	**48 Hours (mm)**
5%	No inhibition	No inhibition
10%	0.5	1.5
50%	1	1.8

**Table 3:** Comparative antimicrobial activity of 50% Extracts at 48 Hours

**Extract Type**	**Zone of Inhibition (mm)**
Mangifera indica	2.7
Pongamia pinnata	1.8

**Table 4:** Summary of antimicrobial activity across concentrations

**Concentration (%)**	**Mango Extract**	**Karanj Extract**
5%	No inhibition	No inhibition
10%	Moderate	Mild
50%	High	Moderate
